# 1993–2014: two decades of predictive testing for Huntington's disease at the Medical Genetics Unit of the University of Genoa

**DOI:** 10.1002/mgg3.238

**Published:** 2017-06-17

**Authors:** Paola Mandich, Merit Lamp, Fabio Gotta, Rossella Gulli, Ariela Iacometti, Roberta Marchese, Emilia Bellone, Giovanni Abbruzzese, Giovanna Ferrandes

**Affiliations:** ^1^ Department of Neuroscience, Rehabilitation, Ophthalmology, Genetics and Maternal‐Child Sciences University of Genoa Genoa Italy; ^2^ Medical Genetics Unit IRCCS AOU San Martino‐IST Genoa Italy; ^3^ Unit of Clinical Psychology and Psychotherapy IRCCS AOU San Martino‐IST Genoa Italy; ^4^ Clinical Neurology Unit IRCCS AOU San Martino‐IST Genoa Italy

**Keywords:** Counseling, Huntington's disease, integrated protocol, predictive testing

## Abstract

**Background:**

Predictive testing for Huntington's disease has been available at the Medical Genetics Unit of the University of Genoa from 1987. In 1989, an integrated counseling protocol (geneticist, psychologist, and neurologist) was developed following International Guidelines.

**Methods:**

This is a retrospective analysis of the clinical charts and motivation questionnaires of persons seeking predictive testing through direct DNA analysis from 1993 until 2014, with the aim to evaluate their individual characteristics, motivations, and the outcomes of the counseling protocol.

**Results:**

A total of 299 persons (164 women, 135 men) applied for predictive testing. Most applicants’ features and motivations were similar to those previously described, but surprisingly the percentage of completed protocols was higher among men, 68.5% versus 53.5% (*P* = 0.011). Likewise, persons over 25 years of age were more likely to take the test than younger applicants (18–25 years): 63.4% versus 48.1% (*P* = 0.043). In addition, relationship status, having children, and the gender of the affected parent showed different effects on the decision about testing in males and females. No catastrophic reactions were reported during the study period.

**Conclusions:**

We observed that factors influencing the decision‐making process might differ between males and females, and that predictive testing appears a safe procedure if framed within an integrated counseling protocol.

## Introduction

Predictive testing (PT) for Huntington's disease (HD) has been available at the Medical Genetics Unit of the University of Genoa since 1987, initially by DNA‐linkage, and since 1993 by direct mutation analysis. Direct testing made family participation no longer necessary, thus providing greater autonomy and privacy to applicants.

PT brings several ethical and practical questions, including the *pros* and *cons* of knowing in advance the future, the respect of the right not‐to‐know, and the consequences of living with the test result.

A protocol for PT has been developed since 1989, at the Medical Genetics Unit, University of Genoa, according to the International Guidelines (World Federation of Neurology: Research Group on Huntington's Chorea, 1989; Went 1990; International Huntington Association [IHA], 1994; MacLeod et al. 2013). The guidelines present four ethical principles according to which the protocol was structured: autonomy, beneficence, nonmaleficence, and justice. Our multidisciplinary team consists of a medical geneticist, a psychologist, and a consultant neurologist. The geneticist and the psychologist receive applicants together; this approach allows to integrate the competence of both specialists and thus a more holistic consideration of the person at risk, which is the basis of the whole counseling process in our protocol. The integrated counseling protocol aims to conjugate the respect of autonomy with maximum benefit, supporting the applicant in the decision making about testing and helping her or him to cope with the results.

This paper reports the sociodemographic characteristics of the PT applicants, their motivations and expectations, and the outcomes of the counseling protocol during two decades of direct HD testing.

## Sample and Methods

### Ethical compliance

This study was approved by the local Ethical Committee and an informed consent was signed by each participant.

This is a retrospective analysis of the clinical charts of persons at risk who came to the Medical Genetics Unit of the University of Genoa for PT for HD from 1993 until 2014. In addition, in the periods between 1994–2000 and 2005–2007, a custom‐made five‐page questionnaire was filled in by most participants at the end of the first counseling session. This questionnaire was used to collect additional information on: the main *pros* and *cons* of PT; the symptoms of HD which were considered the most disabling ones and whether the applicant had lived with an affected relative; expectations about the test results (having/not having inherited the mutation); information about future choices (e.g., reproductive decision making, including prenatal diagnosis and adoption) and the opinion on PT in minors.

The PT protocol consists of at least two pretest counseling sessions and one neurological examination before taking the blood sample for genetic analysis and of one disclosure session (described in detail in Mandich et al. 1998). The main novelty of the PT protocol in Genoa is that both, the geneticist and the psychologist, are in charge of PT and share the ethical responsibility for the test. They are both present during the entire counseling process. Yet, after each visit, the applicant has one vis‐à‐vis session with the psychologist. In one or more sessions, if needed, the psychologist explores several issues with the applicant, for example, life experience with the disease, emotional and cognitive functioning, inner motivation for undergoing the PT, expectations of the test result, autonomy in decision making in order to allow an accurate evaluation of her or his ability to cope with the test result before it is carried out. The pre‐ end posttest sessions are conducted on the basis of nonstructured interview, in order to favor the formation of a trusting relationship between the applicant and the counselors.

The pretest sessions are focused on what the PT result would mean for the applicant and on the impact it might have in order to offer maximum support in the decision‐making process. Additional psychological counseling is provided on a case‐by‐case basis**.**

Short‐ (3 months) and long‐term (6 months, 1 year, 2 year, etc.) follow‐up is offered in each case, but is left optional for the participant. The Patient Health Questionnaire (PHQ‐9), the STAI FORM X‐1, STAI FORM X‐2, and nonstructured interview are used in the follow‐up sessions. All applicants are free to withdraw from the protocol whenever they wish, and can be readmitted by starting again with a counseling session.

Subjects with obvious signs of the disease (UHDRS ≥4, Unified Huntington's Disease Rating Scale [Huntington Study Group, 1996]) are usually referred, in agreement with them, to a neurologist. However, in some cases, symptomatic applicants unaware of their signs are allowed to complete the protocol.

### Data analysis

The data from clinical charts and the questionnaires were analyzed by using PASW 18.0 software (SPSS, Chicago, IL). To identify the factors possibly influencing the decision about taking the test, symptomatic patients who were referred to a neurologist and thus did not complete the protocol were excluded from the analysis. A *P* < 0.05 was considered statistically significant.

## Results

**Figure 1:**
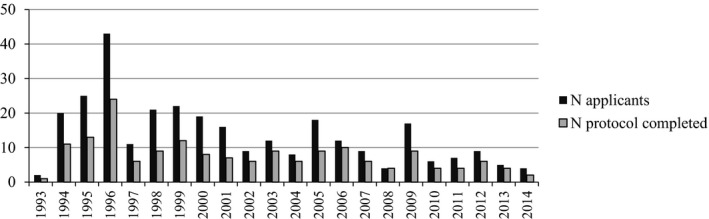
Number of applicants for PT and of tests performed per year from 1993 to 2014.

From December 1993 until December 2014, a total of 299 persons, 164 women (54.8%) and 135 men (45.2%), applied for PT in Genoa. The annual number of applicants for PT and accordingly also the number of tests taken have gradually decreased (see Fig. 1).

The main demographic characteristics and testing outcome are presented in Table 1 along with data from other recent studies on this topic. Average age and the percentage of women in our group was comparable to previous studies, while the proportion of applicants in a stable relationships and of those with children was slightly lower. Also, the number of persons at 50% of risk, the percentage of completed protocols, and of positive test results were among the lower ones. Interestingly, in our group the proportion of men and women who completed the protocol was significantly different: 68.5% versus 53.5% (*P *=* *0.011) (data not shown).

**Table 1:** Demographic data of subjects in the present study and in other recent publications on predictive HD testing

	Present study	Bernhardt et al. (2009)	Panas et al. (2011)	Dufrasne et al. (2011)	Sizer et al. (2012)	Wedderburn et al. (2013)	Scuffham and MacMillan (2014)
Country	Italy	Germany	Greece	Canada	South Africa	Australia (Western)	Australia (Queensland)
Study period	1993–2014	1993–2004	1995–2008	1994–2008	1998–2006	1993–2012	2006–2010
Number of applicants	299	478	256^b^	181	57	466	152
Average age (years)	35.4	35.0	33.8^b^	36.4^b^	30.0	48.9	39.3
Gender: females (%)	54.8	57.0	54.7^b^	61.9 (57.0^b^)	66.7	57.6	54.5
Applicants married or in a stable relationship (%)	66.2	73.8	–	70.4^b^	–	–	70.2
Applicants with children (%)	41.1	44.0	–	57.0^b^	43.9	–	58.5
Applicants with 50% risk (%)	85.3	92.0	–	98.5	–	–	82.3
Protocol completed (%)	56.9 (60.3^a^)	52.0	100.0^b^	74.6	66.7	80.0	62.5
Positive test result (CAG≥36)	37.6	–	48.0	42.2	36.9	37.0	54.6

a Data on 282 applicants after excluding symptomatic patients who were referred to other specialists without taking the test (*n* = 17).

b Data given only about patients who completed the protocol for predictive testing.

The majority of applicants (75.3%) who completed the protocol had three multidisciplinary counseling sessions. The median time between the first counseling session and blood withdrawal was 1.5 months and remained constant during the years. The protocol was modified, prolonged or abbreviated, by the specific needs and motivations of each applicant in order to prevent severe psychological distress after PT outcome. During the pretest period applicants were encouraged to visit or call the psychologist whenever they wished outside the scheduled interviews. When psychological assessment highlighted the risk of clinical distress, the applicant was referred, upon agreement, to an external psychological/psychotherapeutic support.

Applicants who withdrew from PT always did so before blood sampling. Ten applicants, who initially withdrew from the protocol, came back after 2–9 years, mostly to know about their genetic status before planning a pregnancy. Another motivation was the desire to put an end to the uncertainty.

About one third of applicants who took the test (28 males, 28 females; test result positive in 62.5% of cases) returned for long‐term follow‐up sessions. No evidence of major psychological reactions, psychotic episodes, or of attempted suicides was recorded in our PT applicants.

In the period of analysis, 57 young adults (≤25 years; 19.1% of all applicants) applied for PT and 48.1% (26/54; symptomatic patients are excluded) of them completed the protocol, which is significantly less compared with subjects >25 years of age (142/224, 63.4%, symptomatic patients are excluded; *P *=* *0.043). In this subgroup, there was no difference between males and females in taking the test.

When factors possibly influencing decision taking were analyzed, no significant associations were observed with marital and parental status or with the gender of the affected parent. However, when males and females were analyzed separately, some differences emerged (Table 2). Among applicants who were married or in a stable relationship, males were more likely than females to take the test. Likewise, among women and men who already had children, the percentage of completed protocols was significantly lower in females. Among applicants with an affected father, males were more likely than females to take the test.

**Table 2:** Associations between demographic data, gender of the affected parent, and being tested; males compared to females.^a^

	Males *n* tested/total (%)	Females *n* tested/total (%)	*P*‐value
Marital status			
Single	26/45 (57.8)	27/47 (57.4)	ns
Married/stable relationship	60/81 (74.1)	55/105 (52.4)	0.003
Separated/divorced/widowed	1/1 (100)	1/3 (33.3)	ns
Children			
Yes	26/37 (70.3)	35/76 (46.1)	0.015
No	56/83 (67.5)	47/76 (61.8)	ns
Expecting a child	5/7 (71.4)	1/3 (33.3)	ns
Affected parent			
Mother	37/57 (64.9)	41/69 (59.4)	ns
Father	37/52 (71.2)	26/55 (47.3)	0.012

a Symptomatic patients who were referred to other specialists (neurologist/psychiatrist) without taking the test (*n* = 17) are excluded; ns, not significant.

### Questionnaire

The questionnaire was filled in by 120 applicants. The motivations for PT or reasons to decline PT are given in Table 3. The main motivation for testing was the “need to know/reduce the uncertainty,” while the primary reason against taking the test was the “difficulty to accept a positive test result.” There was a significant difference in reasons against testing between applicants who had lived with an affected relative and those who had not, as the latter mentioned “negative effects on family relationships” significantly more often (29.4% vs. 10.5%, *P *=* *0.037). Only two participants, who withdrew from the process, indicated the “complexity of the protocol” as a potential reason for not taking the test.

**Table 3:** Main motivations in favor and against predictive testing

	Total (*n* = 120)	Gender			Protocol completed			Lived with an affected relative		
		Males (*n* = 50)	Females (*n* = 70)	*P*‐value	Yes (*n* = 70)	No (*n* = 50)	*P*‐value	Yes (*n* = 86)	No (*n* = 17)	*P*‐value
Motivations in favor of testing										
Need to know	78 (65.0)	35 (70.0)	43 (61.4)	ns	50 (71.4)	28 (56.0)	ns	54 (62.8)	13 (76.5)	ns
Informing children	58 (48.3)	23 (46.0)	35 (50.0)	ns	34 (48.6)	24 (48.0)	ns	44 (51.2)	7 (41.2)	ns
Planning the future	52 (43.3)	22 (44.0)	30 (42.9)	ns	34 (48.6)	18 (36.0)	ns	41 (47.7)	6 (35.3)	ns
Family planning	51 (42.1)	18 (35.3)	33 (47.1)	ns	30 (42.3)	21 (42.0)	ns	38 (44.2)	9 (52.9)	ns
Motivations against testing										
Difficulty accepting positive results	41 (34.2)	19 (38.0)	22 (31.4)	ns	22 (31.4)	19 (38.0)	ns	23 (26.7)	4 (23.5)	ns
Absence of therapy	26 (21.7)	7 (14.0)	19 (27.1)	ns	15 (21.4)	11 (22.0)	ns	19 (22.1)	6 (35.3)	ns
Negative effect on family relationships	14 (11.7)	5 (10)	9 (12.9)	ns	8 (11.4)	6 (12.0)	ns	9 (10.5)	5 (29.4)	0.037
Protocol complexity	2 (1.7)	0	2 (2.9)	ns	0	2 (4.0)	ns	1 (1.2)	1 (5.9)	ns

ns, not significant.

Table 4 outlines the symptoms that the applicants considered the most disabling ones in their relatives. Involuntary movements and loss of self‐sufficiency were the symptoms mostly reported. Change in personality and depression were reported more frequently by females.

**Table 4:** HD symptoms and signs observed in affected relatives and considered the most disabling ones

Symptom/Sign	Total (*n* = 120)	Gender			Protocol completed			Lived with an affected relative		
		Males (*n* = 50)	Females (*n* = 70)	*P*‐value	Yes (*n* = 70)	No (*n* = 50)	*P*‐value	Yes (*n* = 86)	No (*n* = 17)	*P*‐value
Involuntary movements	102 (85.0)	43 (86.0)	59 (84.3)	ns	58 (85.3)	44 (84.6)	ns	72 (83.7)	15 (93.8)	ns
Loss of self‐sufficiency	56 (46.7)	20 (40.0)	36 (51.4)	ns	35 (51.5)	21 (40.4)	ns	47 (54.7)	8 (50.0)	ns
Personality changes	37 (30.8)	10 (20.0)	27 (38.6)	0.030	22 (32.4)	15 (28.8)	ns	32 (37.2)	3 (18.8)	ns
Aggressiveness	34 (28.3)	10 (20.0)	24 (34.3)	ns	23 (33.8)	11 (21.2)	ns	31 (36.0)	2 (12.5)	ns
Depression	29 (24.2)	7 (14.0)	22 (31.4)	0.028	17 (25.0)	12 (23.1)	ns	24 (27.9)	2 (12.5)	ns
Cognitive decline	27 (22.5)	12 (24.0)	15 (21.4)	ns	17 (25.0)	10 (19.2)	ns	19 (22.1)	4 (25.0)	ns

ns, not significant.

The majority of applicants (75.2%) had no premonition of whether they had inherited the mutation or not, but the proportion of females who believed to be mutation carriers (13 of 68 who answered that question, 19.1%) was significantly larger than in males (1 of 49, 2.0%; *P *=* *0.010). In the group of young adults, 73.3% had no definite expectation of test result without gender differences.

Most of the participants (74.0%) declared that they would ask for prenatal testing, if they would prove to have the HD mutation. However, during the study period, only 11 (17.2%) of those who proved to be carriers, returned for prenatal testing.

A minority of all applicants (16.7%) were in favor of predictive testing in minors and, among young adults, this percentage was even lower (9.5%).

### Symptomatic applicants

Of all the individuals who applied for PT, 33 persons (11.0% of all applicants) presented already minor signs or symptoms which could be associated with HD. Seventeen of them, aware of their signs, agreed to be referred to a neurologist without taking the test, six decided autonomously not to proceed with testing while 10 applicants, unaware of the possible disease onset, were allowed to continue with the protocol (test result was positive in all cases). The mean age of symptomatic patients was significantly higher than that of asymptomatic persons: 41.5 versus 34.7 years, respectively (*P *=* *0.001).

### Overall evaluation of the PT protocol

In general, applicants who returned for long‐term follow‐up expressed during the interview their satisfaction with the whole PT protocol. The team was perceived as a source of support during the whole protocol. The multidisciplinary approach and the pretest counseling sessions were considered of great value as it offered applicants the opportunity to deeply explore their motivation to undergo the test.

## Discussion

Previous studies have reported that the majority of applicants for PT undergo the DNA test for HD (Trembath et al. 2006; Dufrasne et al. 2011; Sizer et al. 2012; Wedderburn et al. 2013; Scuffham and MacMillan 2014). Data from Italy during the period 1994–1996 showed that only 49% of applicants completed the PT protocol (Mandich et al. 1998). In the present study, this percentage was somewhat higher (60.3%) and comparable to other populations (Table 1) if symptomatic applicants who did not complete the protocol were excluded.

Similarly to other studies, the request for PT in our center has decreased over time (Trembath et al. 2006; Bernhardt et al. 2009; Creighton et al. 2003; Panas et al. 2011). It is likely that in the first years of direct CAG testing the number of applicants was higher, since at‐risk individuals who had declined linkage analysis because of the need for family participation could now proceed with direct testing.

### Gender dissimilarities

An overrepresentation of females among those who seek predictive testing is reported worldwide (Trembath et al. 2006; Dufrasne et al. 2011; Sizer et al. 2012; Wedderburn et al. 2013; Scuffham and MacMillan 2014; Bernhardt et al. 2009; Panas et al. 2011; Hayden 2000; Rodrigues et al. 2012) and this was confirmed also by our study. However, if we look at the percentage of those who completed the protocol, it was significantly higher among males. A similar trend was reported previously by Dufrasne et al. (2011), although it was not highlighted or discussed in detail. Furthermore, in our cohort, women living in a stable relationship and/or having children abandoned the test protocol more often than the matching group of male applicants. We can only hypothesize that these differences may be based on gender role dissimilarities in the Italian population. In Italy women still have a prevailing role in caregiving activities compared to men (Banca d'Italia, 2000; Istat, 2000, 2010). Moreover, women are in general more relationship‐oriented and therefore probably more influenced by the condition of their partner than men (Lyons et al. 1995). It is thus possible that women's greater involvement, both physical and emotional, in caregiving encourages married male applicants to expect a stable and adequate assistance by their partners over time, reassuring them to complete the test protocol. Another hypothesis is that women, as main caregivers, may be more anxious about the possible negative impact of a positive test result on their psychological and physical well‐being and its subsequent effect on the family's everyday life. Likewise, women's greater involvement in children care may be one of the reasons for not wanting to undergo the PT, since worry and distress associated with a carrier status could represent a heavy burden and might compromise their protective and educational role with children. On the other hand, it is possible that the partners of at‐risk women are the ones worried about the possible negative impact of the test result and thus might persuade women not to take the test.

Another difference that we observed between female and male applicants in completing the PT protocol was related to the gender of the affected parent as men with an affected father were more likely to take the test. This result is opposite to what was reported in previous studies, where men with an affected mother were more likely to complete the protocol (Trembath et al. 2006; Scuffham and MacMillan 2014; Goizet et al. 2002; Arning et al. 2015). The reasons for these discrepancies might again lie in the different gender roles in the Italian family context, including the stereotyped role of males in financial wellbeing of the family, and also men's identification with their father.

The main reasons for/against the test reported by applicants of this study are comparable with those reported previously (Dufrasne et al. 2011; Scuffham and MacMillan 2014; Evers‐Kiebooms and Decruyenaere 1998; van der Steenstraten et al. 1994), without significant differences between study subgroups. The only exception was that applicants who had not lived with an affected relative mentioned “negative effects on family relationships” as a reason against testing significantly more often than those who had lived with an affected relative. Perhaps a previous experience with an affected relative could result in learning that fosters the perception of self‐efficacy, which in turn helps to form expectations of greater control over future challenges (Bandura 1997).

Women reported more frequently “change of personality” and “depression” as the most disabling HD symptoms. This finding may suggest that women perceive behavioral symptoms of HD as the most difficult to manage due to the fact that women are the ones who most often have close relationships with patients and take care of them, as reported above.

Most applicants’ features in the present study were overlapping with those previously described. The few differences we observed in our cohort may be related to the population's characteristics (Italy vs. other countries) and/or the protocol used. The gender differences we observed may deserve further investigation in other populations with a different cultural background. If the applicant's gender plays a role in the decision‐making process, knowing it and taking it into consideration might be useful for improving the counseling process and PT protocols’ outcome.

### Expectations about the test results (having/not having inherited the mutation)

We observed a significant difference between males and females in the proportion of those who believed to be mutation carriers. This corresponds with previous findings that more females than males anticipated an unfavorable test outcome (Tibben et al. 1993a). Most young adults had no definite expectation of test result in contrast to a previous study, where most of them believed to be mutation carriers (MacLeod et al. 2014). However, these findings are not easily comparable due to differences in sample size and the timing, pre‐ or posttest, of data collection.

### Symptomatic applicants

The number of PT requests by symptomatic individuals was unexpectedly high. About half of them were referred to neurologists or psychiatrists, while individuals unaware of their illness, were allowed to continue the protocol in order to allow them to prepare psychologically for the HD diagnosis and to help them accept it gradually. This approach, initially proposed by Tibben (2007), has revealed to be fruitful also in our experience. In our opinion, it is important to offer symptomatic individuals who lack insight into their symptoms or deny having symptoms, the time to get aware of the beginning disease process, and for expressing frailty and fears in order to allow them to recognize and empower their inner resources for coping. Therefore, the integrated genetic counseling process represents, for symptomatic individuals, a helpful preliminary experience for dealing with the HD diagnosis.

### Young adult applicants

Predictive testing in minors for late onset conditions without possibilities of therapy or of clinical intervention for modifying disease progression and prognosis was addressed in International Guidelines (MacLeod et al. 2013). There is no consensus between countries, according to the Local Law Codes, about the minimum age that must be considered as the limit between minors and adults (14–18 years) for access to predictive testing.

For many reasons the attitudes and expectations of minors and young adults about PT have been rarely explored. In our sample, there were no differences among young adults and other applicants in reasons for/against PT. Nevertheless, a lower percentage of young adults completed the protocol compared with older applicants. A possible explanation for this difference may be related to the motivations reported in favor of testing. Most likely family planning and informing children are not yet a priority for young adults.

A previous study reported that nearly half of applicants and their partners thought that minors should be free to undergo PT (Tibben et al. 1993b). In our study, instead, the majority of participants were not in favor of lowering the age of testing, including young adults, which is in agreement with the data reported elsewhere (MacLeod et al. 2014).

### Prenatal diagnosis request

In contrast to what was stated by applicants in the pretest questionnaire, the number of actual requests for prenatal diagnosis by mutation carriers was very low over the years. There might be several explanations for this phenomenon (Schulman and Stern 2015), but exploring them in detail was beyond the scope of the present study and therefore will not be discussed here. Future studies should be done on this subject.

## Conclusions

This is a retrospective analysis of data gathered on persons seeking PT during 20 years of practice with the aim to evaluate their individual characteristics, motivations, and the outcomes of the counseling protocol.

Overall the PT can be considered a safe procedure if performed in the context of a sound and flexible multidisciplinary tailored counseling. In our experience, combining medical genetics and psychological expertise is an effective approach to take care of applicants during the PT and to help them make conscious choices.

In conclusion, we observed that factors influencing the decision‐making process might differ between males and females, and that PT appears a safe procedure if framed within an integrated counseling protocol.

## Conflict of Interest

None declared.
